# Application of composite layered repair system using titanium mesh, bone cement and vacuum sealing drainage for chest wall defect

**DOI:** 10.3389/fmed.2025.1681418

**Published:** 2025-10-30

**Authors:** Gaofeng Liu, Xiaoyong Ding, Qingyuan Li, Xia Chu, Yingbiao Deng, Sujuan Cui, Li Zhou, Chaofeng Xing, Xiaohang Sun, Jie Zhang

**Affiliations:** ^1^The 988th Hospital of Joint Logistics Support Forces, PLA, Zhengzhou, Henan Province, China; ^2^Beijing North Medical District, PLA General Hospital, Beijing, China

**Keywords:** chest wall defect, titanium mesh, bone cement, vacuum sealing drainage, reconstruction

## Abstract

**Objective:**

To evaluate the therapeutic efficacy of a composite layered repair system utilizing titanium mesh (TM), bone cement (BC) and vacuum sealing drainage (VSD) for chest wall defect reconstruction, providing a reliable theoretical foundation and practical guidance for clinical chest wall trauma management.

**Method:**

A thoracic defect model (≥6 × 6 cm^2^) was established in 22 adult goat and then divided into the TM + BC + VSD, TM + BC and TM + VSD groups. The survival status, activity status, wound recovery, vital signs, blood routine, arterial blood gas and inflammatory factor levels of the three goat groups were monitored and compared after the operation.

**Result:**

All groups demonstrated comparable survival rates and periods with satisfactory defect repair outcomes. The PaO_2_ at multiple time points after surgery in the TM + BC + VSD group were higher than those in the TM + BC and TM + VSD groups, while the activity status score, WBC and levels of PaCO_2_, IL-2, IL-6, IL-10, IL-17 and TNF-*α* were lower than those in the TM + BC and TM + VSD groups. The SpO_2_, hemoglobin and erythrocyte were higher than those in the TM + VSD group, and the wound healing score, heart rate was lower than that in the TM + VSD group.

**Conclusion:**

The composite laminated repair system constructed by TM, BC and VSD can increase the survival rate after chest wall defect repair, promote functional recovery, improve oxygenation and reduce inflammatory responses, and has potential clinical application value.

## 1 Introduction

Chest wall defect refers to the destruction of the bony structure and/or soft tissue integrity of the chest wall caused by trauma, tumor resection, infection or congenital factors, which is a major challenge faced in the clinical practice of trauma surgery and thoracic surgery (1). From an anatomical perspective, the chest wall is a complex composed of bony structures and soft tissues, and it has important functions such as protecting internal organs and maintaining respiratory movements (2). When the defect range of the chest wall exceeds 5 cm (anterior chest wall) or 10 cm (posterior chest wall), especially when more than three ribs or sternum are involved, it will seriously affect the integrity of the thoracic cage, leading to serious complications such as abnormal breathing, respiratory dysfunction, and even endangering life (3, 4). Therefore, the treatment, repair and reconstruction of chest wall defects have important clinical significance.

Chest wall reconstruction includes bony reconstruction and soft tissue reconstruction, that is, the integrity of the bony structure. Once the stability of the chest wall is restored, the coverage of soft tissues and the free movement of flaps will eventually complete the chest wall reconstruction (5). At present, the materials for reconstructing chest wall defects are classified into three types: autologous tissues, allogeneic tissues and artificial materials. However, a single built-in material has many limitations. Therefore, some scholars have used Hybrid technology to reconstruct the bony thoracic cage and then applied patent-like materials to repair closed chest wall defects. Among them, the “sandwich” composite material containing bone cement (BC) has significant advantages (6, 7). However, it still has disadvantages such as limited chest wall movement after reconstruction, possible local effusion, infection or loosening of the support in the complex, and inability to be applied in the case of infection. Therefore, exploring a bony thoracic cage reconstruction method that is more in line with physiology is the research focus in thoracic surgery.

In recent years, significant progress has been made in chest wall reconstruction techniques. Previous studies have found that titanium mesh (TM) can maintain the integrity and stability of the bony thoracic cage in repairing chest wall defects caused by diseases such as chest wall tumors, and prevent chest wall collapse and abnormal breathing (8–10). In addition, filling the bone defect area with BC stimulates the formation of the induced membrane. The induced membrane can secrete various bone growth factors and adsorb BMSCs and has rich microvessels. It also has the function of isolating and wrapping the bone graft material and has a good bone repair effect (11, 12). Vacuum sealing drainage (VSD) covering the wound surface of soft tissue defects is beneficial for controlling local infection, improving local blood circulation, and promoting the growth of granulation tissue (13). However, when these single materials or techniques are applied to the repair of chest wall defects, there are obvious limitations. Although TM can effectively maintain the stability of the thoracic cage, its mesh structure may lead to open pneumothorax and limited control ability over wound infection (8). Although BC has good anti-infection properties, it lacks sufficient mechanical strength and cannot solve the problem of chest wall floating. Although the VSD technique can achieve wound closure and adequate drainage, it is powerless against chest wall collapse caused by bony thoracic defects. Therefore, this study proposes a composite laminated repair scheme combining TM, BC and VSD technology, aiming to explore the influence of different repair materials and methods on the reconstruction of chest wall defects after chest wall trauma, to provide the experimental basis for subsequent chest trauma management.

## 2 Materials and methods

### 2.1 Materials

Twenty-one healthy adult goat (aged 18–25 months, weighing 15–50 kilograms) with normal vital signs and no history of injury or medication were provided by the Fuping County Branch of Shaanxi Junxing Biotechnology Co., LTD. The experimental animal production license and usage permit number is SCXK (Shaanxi) 20180082. After 1 week of adaptive feeding, the animals were provided with free access to feed and water throughout the experimental period. This study was approved by the Institutional Ethics Committee (Approval No. 988YY20230001LLSP).

Surgical cabin supporting Equipment (2007-MFH) provided by the General Armament Department of the People’s Liberation Army of China; TM (150 × 150 mm) was purchased from Zhengzhou Meisen Medical Devices Co., LTD, China; BC (Palacos R + G(antibiotics)) was purchased from Heraeus Medical GmbH; VSD auxiliary material set (NPQ-FL-15 × 15) was purchased from Hunan Depus Medical Devices Co., LTD.

### 2.2 Establishment of an animal model of chest wall defect

**Figure 1:**
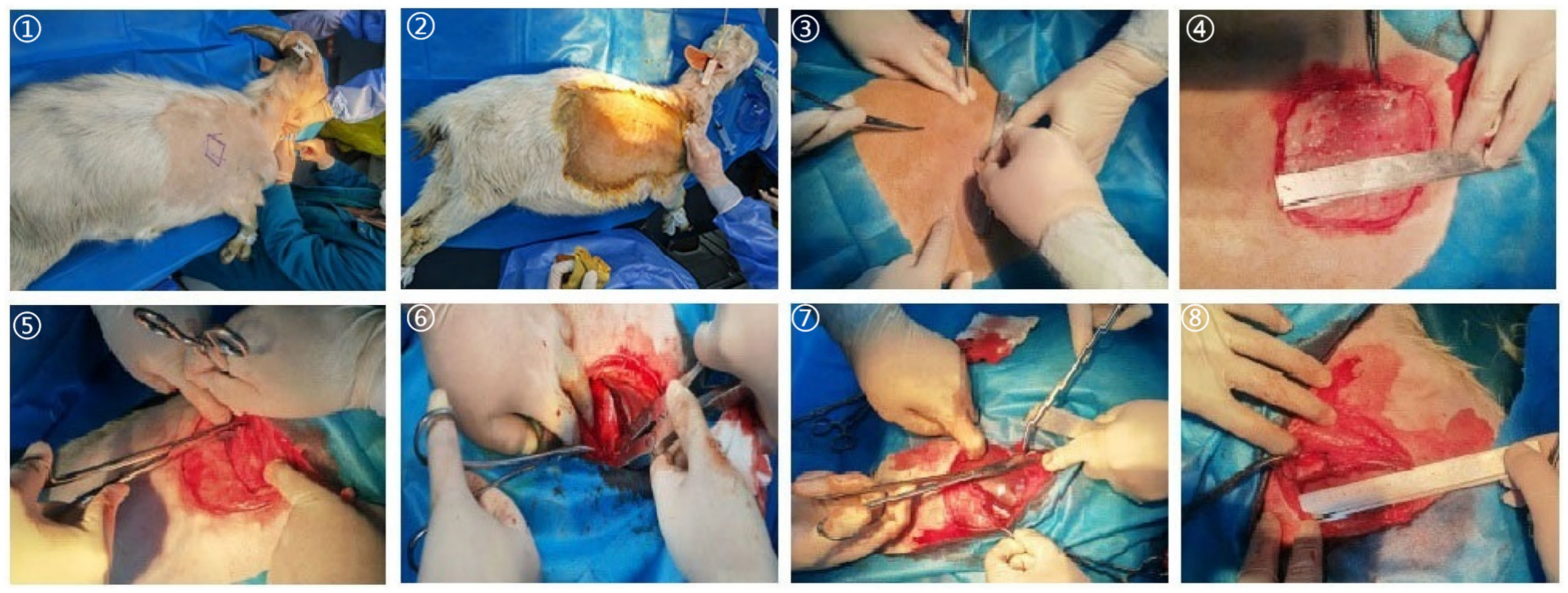
Establishment of an animal model of chest wall defect.

Following general anesthesia, the right thoracic wall tissue of each goat was resected to prepare a thoracic defect model with a size of no less than ≥6 × 6 cm^2^. The specific steps are as follows (Figure 1): (1) Skin disinfection, skin cutting, separation of fat, muscle and other tissues, and exposure of the 5th to 8th ribs; (2) Measure the size of the defect with a sterile ruler; (3) Use the bone-biting forceps to bite off the 5th to 8th ribs, resect the bitten ribs and the adjacent intercostal muscles, and prepare chest wall defects no less than 6 × 6 cm^2^; (4) When removing the ribs, ligate the intercostal arteries and apply bone wax to the bone marrow cavity.

### 2.3 Reconstruction of chest wall defect

**Figure 2:**
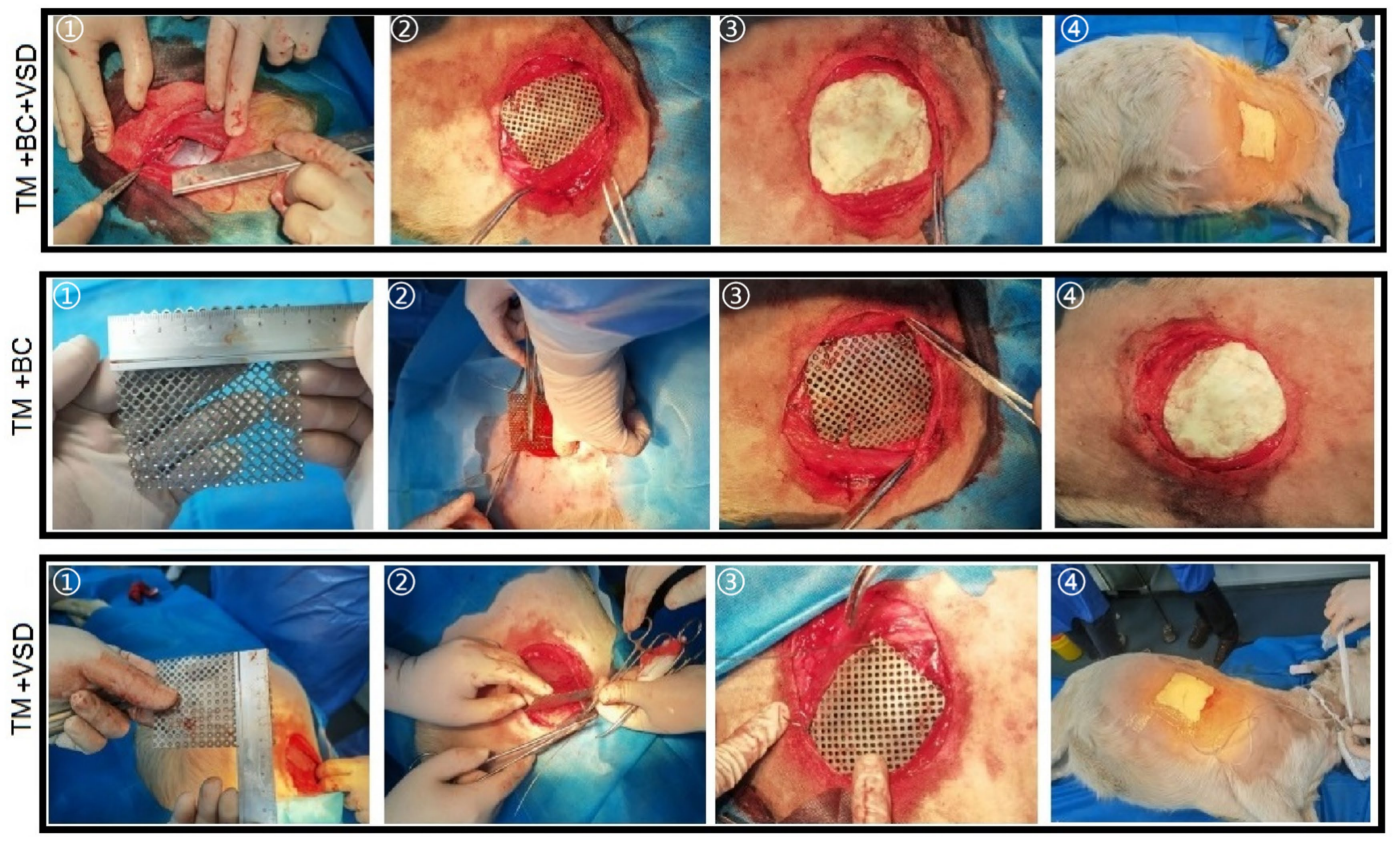
Reconstruction of chest wall defects.

Twenty-one goat were randomly divided into the TM + BC + VSD, TM + BC and TM + VSD groups by random lottery, with 7 goat in each group. The steps for repairing chest wall defects in goat in the TM + BC + VSD group (Figure 2): (1) TM fixation: Measure the size of the chest wall defect, cut an appropriate TM for repair, and fix the TM to the ribs with steel wire. First, fix the right anterior lower rib and the TM, then the right anterior upper rib and the TM, followed by the right posterior lower rib and the TM, and finally the right posterior lower rib and the TM. Finally, use No. 7 silk thread to fix the right anterior and right posterior intercostal muscles and the TM. (2) BC sealing: Then, evenly cover the TM with the adjusted BC, with a thickness of 3 mm to 5 mm. (3) Establish VSD: Cover the surface of the BC with VSD excipients, stick and fix them with medical transparent adhesive tape excipients, connect a portable VSD negative pressure device, adjust the continuous negative pressure suction mode, and adjust the pressure to 50–100 mmHg. Fix the VSD negative pressure device to the neck of the goat with a bandage. (4) After the operation, ceftizoxime at a dose of 5 mg/kg and 20 mL of normal saline were intravenously injected. The repair of chest wall defects in goat in the TM + BC group (Figure 2) was the same as that in the TM + BC + VSD group except for not establishing VSD. The repair of chest wall defects in goat in the TM + VSD group (Figure 2) was the same as that in the TM + BC + VSD group except for not performing BC sealing. After the operation, the operation records were completed. After the goat regained consciousness from anesthesia, they were observed for 2 h to monitor vital signs such as heart rate, respiration, body temperature, and blood oxygen saturation. Once they were stable, they were sent to the breeding farm for rearing for a total of 28 days.

### 2.4 Observational index

Postoperative recovery and vital sign monitoring: The activity status, wound recovery, and vital signs (including heart rate, body weight, body temperature and blood oxygen saturation) of the goat in all three groups were monitored at the following time points: preoperation (T0), and post-operation days 1, 3, 5, as well as weeks 1, 2, 3, and 4 (T1–T7). Chest wall repair was assessed via digital radiography (DR). The effect of composite laminated materials on chest wall injury repair was preliminarily evaluated through radiographic analysis. Activity status score: 1 point: active and with a normal daily food intake; 2 points: Mainly standing but with reduced activity level and range, and daily food intake reduced by one-third compared to normal; 3 points: Mainly lying down, stands up after sound stimulation or physical contact, and daily food intake reduced by half compared to normal; 4 points: Weak, drowsy, responsive to sound stimulation and physical touch but unable to stand, only drinking a small amount of water. Wound recovery score: 1 point: Dry wound with no exudate; 2 points: Wound exudate of 5–10 mL without an unpleasant odor; 3 points: Wound exudate ≥10 mL, accompanied by an unpleasant odor and signs of infection; 4 points: Purulent discharge with a foul smell, and persistent infection symptoms for more than 3 days. (2) Blood gas tests: Arterial partial pressure of oxygen (PaO_2_) and partial pressure of carbon dioxide (PaCO_2_) were measured using a blood gas analyzer, and oxygen saturation (SaO_2_) was monitored via pulse oximetry at all time points (T0–T7). (3)Routine blood and biochemical indicators: Animal blood samples were collected at T0–T7 respectively, and hemoglobin, red blood cells, platelets and white blood cell (WBC) were detected by the fully automatic blood cell analyzer BC-5390, and serum alkaline phosphatase (ALP), blood calcium and blood phosphorus were detected by the fully automatic biochemical analyzer. (4) Serum inflammatory factors: The levels of interleukin-6 (IL-6), IL-2, IL-10, IL-17, IL-4, IL-12p70, tumor necrosis factor-*α* (TNF-α), and interferon-*γ* (IFN-γ) at T0–T7 were quantified using ELISA kits.

### 2.5 Statistical analysis

Data were analyzed and plotted using SPSS26.0. The measurement data were presented as mean ± SD. One-way analysis of variance was used for comparisons among multiple groups, and the LSD method was used for pairwise comparisons afterward. Counting data were described by the number of cases (%), and the chi-square test was used for comparison among multiple groups. For repeated measurement data, repeated measures analysis of variance was used for analysis, and multiple comparisons were conducted using the LSD method. *p* < 0.05 was considered statistically significant.

## 3 Result

### 3.1 General information comparison among the three goat groups

There was no statistically significant difference in the general data among the three goat groups (*p* > 0.05, Table 1).

**Table 1:** General information comparison among the three goat groups.

Group	*n*	Weight (kg)	Age (month)	Heart rate (times/min)	Skin temperature (°C)	SpO_2_ (%)
TM + BC + VSD	7	29.87 ± 5.89	22.0 ± 2.00	70.00 ± 6.81	36.4 ± 0.37	98.0 ± 0.81
TM + BC	7	29.16 ± 5.56	21.0 ± 2.47	75.71 ± 8.59	36.6 ± 0.41	98.0 ± 0.95
TM + VSD	7	31.98 ± 6.18	22.0 ± 2.00	73.43 ± 7.80	36.8 ± 0.46	98.0 ± 0.82
*F* value		0.357	0.388	0.923	1.606	0.255
*p* value		0.704	0.684	0.415	0.236	0.777

### 3.2 Postoperative recovery comparison among the three goat groups

**Figure 3:**
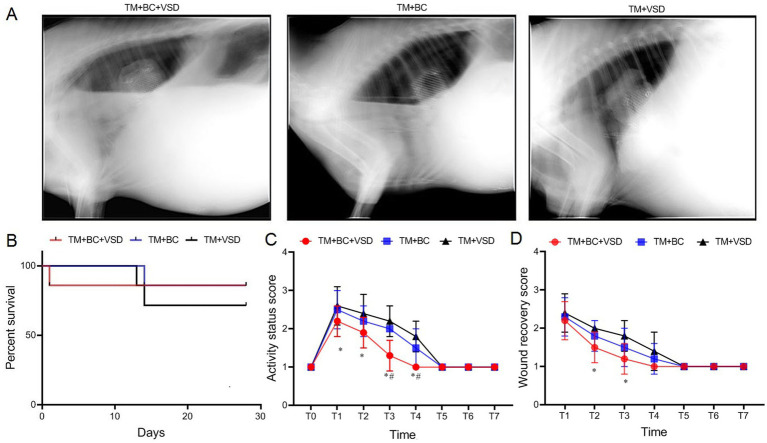
Postoperative recovery comparison among the three goat groups. **(A)** Portable DR imaging of Chest wall defect repair; **(B)** Postoperative survival comparison among the three groups; **(C)** Postoperative activity status score comparison among the three groups; **(D)** Postoperative wound recovery score comparison among the three groups. Compared with the TM + VSD group, ^*^p < 0.05; Compared with the TM + BC group, ^#^*p* < 0.05.

The portable DR detection results revealed that all three repair methods had good repair effects (Figure 3A). At the end of the breeding period, 17 goat survived (with a survival rate of 80.9%). Among them, 6 goat (85.7%) survived in the TM + BC + VSD group and TM + BC group and 5 goat (71.4%) survived in the TM + VSD group. There were no statistically significant differences in the survival rate and survival period among the three goat groups (*p* > 0.05, Figure 3B).

There were no statistically significant differences in the preoperative activity status score among the three goat groups (*p* > 0.05, Figures 3C,D). The activity status scores first increased and then decreased after the operation, peaking at T1 (*p* < 0.05, Figures 3C, D). The activity status scores of the TM + BC + VSD group at T1–T4 were significantly lower than those of the TM + VSD group, and the scores at T3–T4 were significantly lower than those of the TM + BC group (*p* < 0.05, Figure 3C). The wound recovery scores among the three goat groups continued to decrease from T1 onward. The wound recovery scores of the TM + BC + VSD group at T2–T3 were significantly lower than those of the TM + VSD group (*p* < 0.05, Figure 3D).

### 3.3 Vital signs comparison among the three goat groups

**Figure 4:**
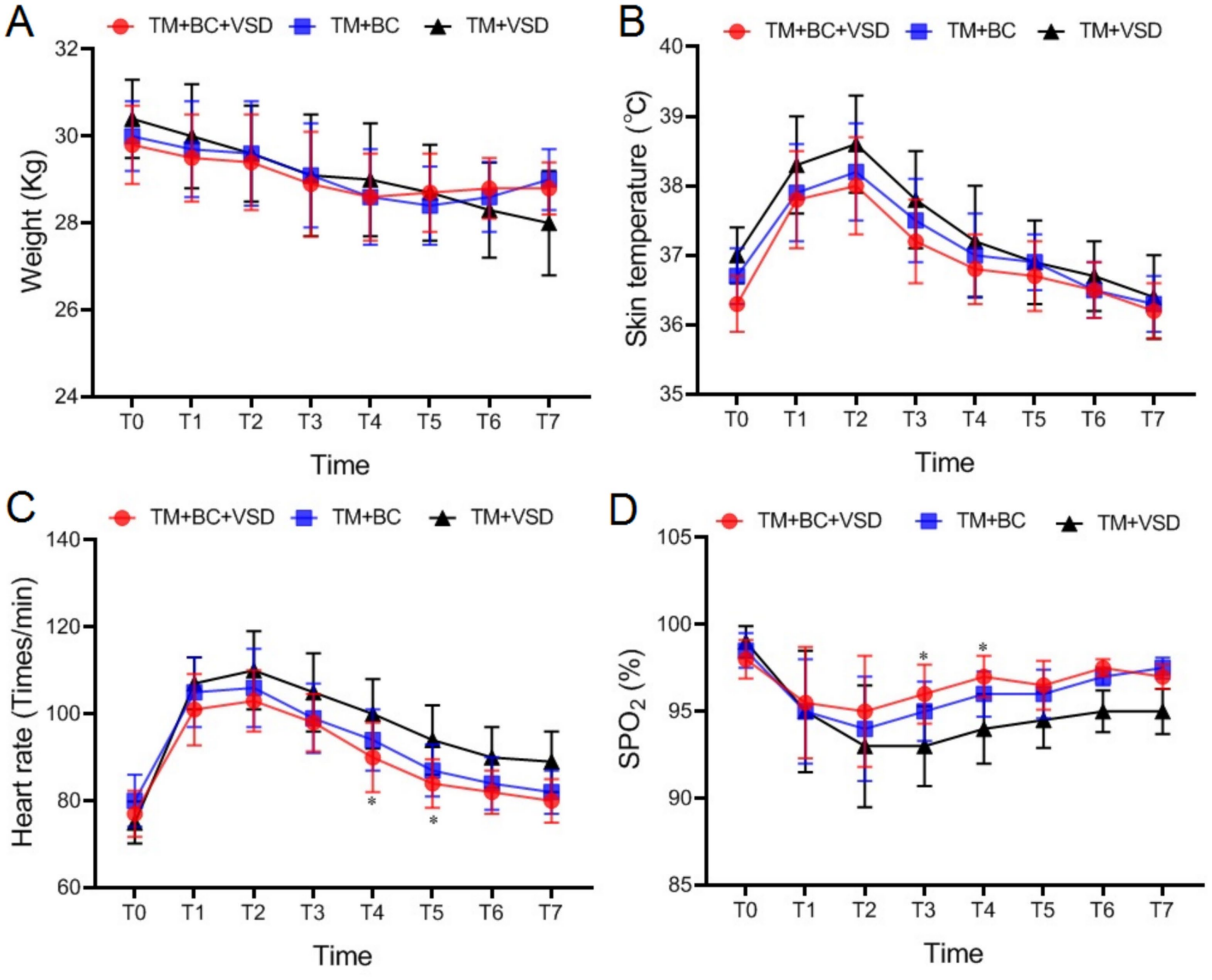
Vital signs comparison among the three goat groups. **(A)** Weight comparison among the three groups; **(B)** Skin temperatures comparison among the three groups; **(C)** Heart rates comparison among the three groups; **(D)** SpO_2_ comparison among the three groups; Compared with the TM + VSD group, ^*^p < 0.05; Compared with the TM + BC group, ^#^*p* < 0.05.

There were no statistically significant differences in preoperative body weight, skin temperature, heart rate and SpO_2_ among the three goat groups (*p* > 0.05, Figure 4). After the operation, body weight decreased, skin temperature and heart rate initially increased and then decreased. Conversely, SpO_2_ decreased and then increased (*p* < 0.05, Figure 4). The heart rate at T4–T5 in the TM + BC + VSD group was significantly lower than that in the TM + VSD group. The SpO_2_ at T3–T4 was significantly higher than those in the TM + VSD group. No statistically significant differences were observed in body weight among the three groups (*p* > 0.05, Figure 4).

### 3.4 Routine blood and biochemical indicators comparison among the three goat groups

**Figure 5:**
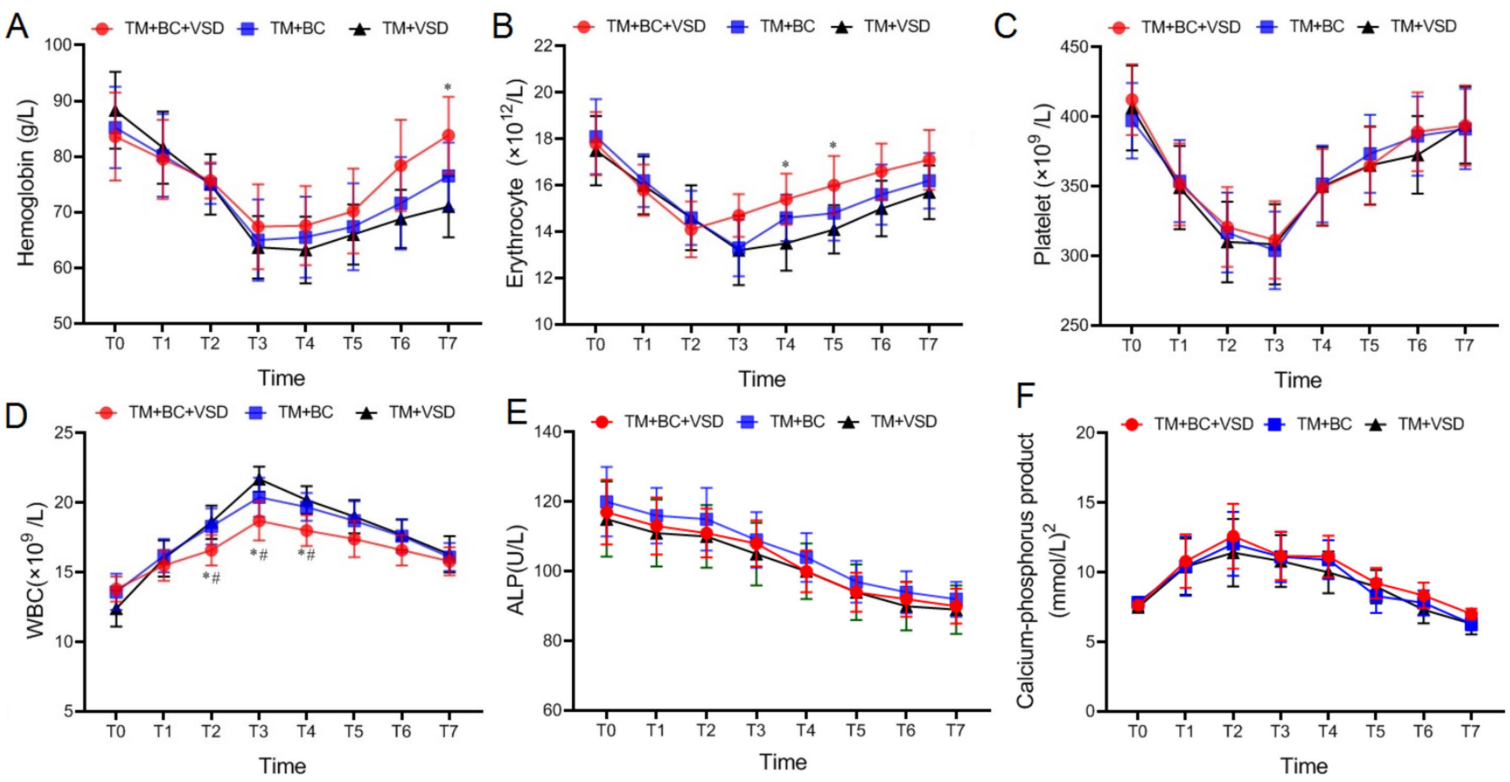
Routine blood and biochemical indicators comparison among the three goat groups. **(A)** Hemoglobin comparison among the three groups; **(B)** Erythrocyte comparison among the three groups; **(C)** Platelet comparison among the three groups; **(D)** WBC comparison among the three groups; **(E)** ALP comparison among the three groups; **(F)** Calcium-phosphorus product comparison among the three groups. Compared with the TM + VSD group, ^*^p < 0.05; Compared with the TM + BC group, ^#^*p* < 0.05.

There were no statistically significant differences in preoperative hemoglobin, erythrocyte, platelet, WBC, ALP and calcium-phosphorus product among the three goat groups (*p* > 0.05, Figure 5). After the operation, the APL continued to decrease, WBC levels and calcium-phosphorus product initially increased and then decreased. Conversely, hemoglobin, erythrocyte and platelet initially decreased and then increased (*p* < 0.05, Figure 5). The hemoglobin at T7 and the erythrocyte at T4–T5 in the TM + BC + VSD group were significantly higher than those in the TM + VSD group. The WBC at T2–T4 in the TM + BC + VSD group and calcium-phosphorus product was significantly lower than that in both the TM + VSD and TM + BC groups (*p* < 0.05, Figure 5). No statistically significant differences were observed in the remaining indicators among the three groups (*p* > 0.05, Figure 5).

### 3.5 Blood gas indicators comparison among the three goat groups

**Figure 6:**
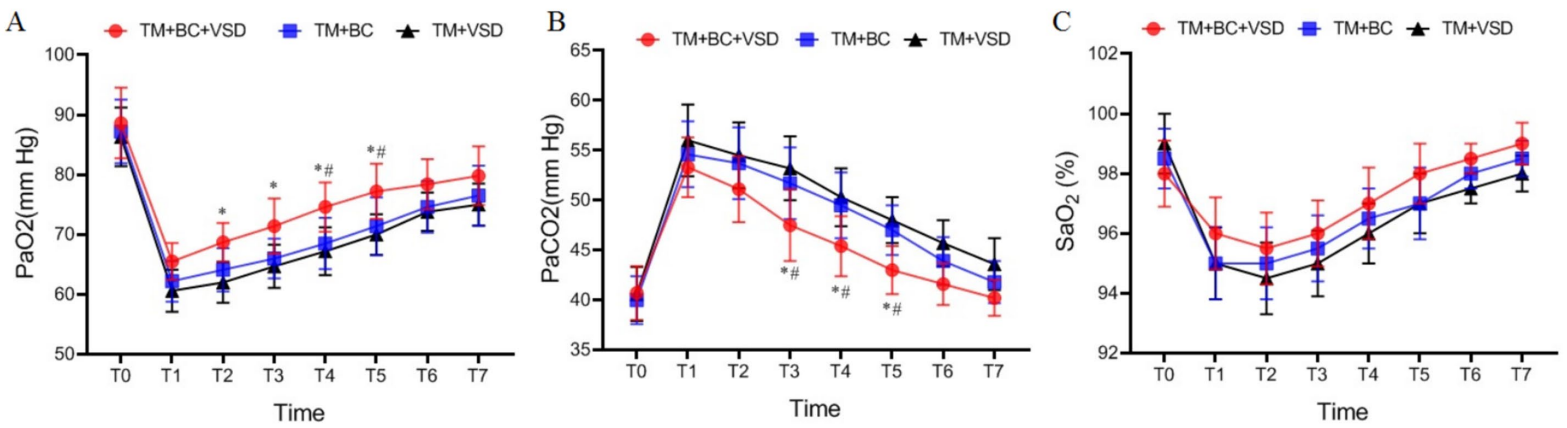
Blood gas indicators comparison among the three goat groups. **(A)** PaO_2_ comparison among the three groups; **(B)** PaCO_2_ comparison among the three groups; **(C)** SaO_2_ comparison among three groups. Compared with the TM + VSD group, ^*^p < 0.05; Compared with the TM + BC group, ^#^*p* < 0.05.

There were no statistically significant differences in the levels of PaO_2_, PaCO_2_ and SaO_2_ among the three goat groups before the operation (*p* > 0.05, Figure 6). After the operation, the levels of PaO_2_ and SaO_2_ initially decreased and then increased, while PaCO_2_ initially increased and then decreased (*p* < 0.05, Figure 6). The PaO_2_ in the TM + BC + VSD group at T2–T5 was significantly higher than that in the TM + VSD group, the PaO_2_ at T3–T4 was significantly higher than that in the TM + BC group (*p* < 0.05, Figure 6). The PaCO_2_ at T3–T5 was significantly lower than that in both the TM + VSD and TM + BC groups (*p* < 0.05, Figure 6). No statistically significant differences were observed in SaO_2_ among the three groups (*p* > 0.05, Figure 6).

### 3.6 Inflammatory factor levels comparison among three goat groups

**Figure 7:**
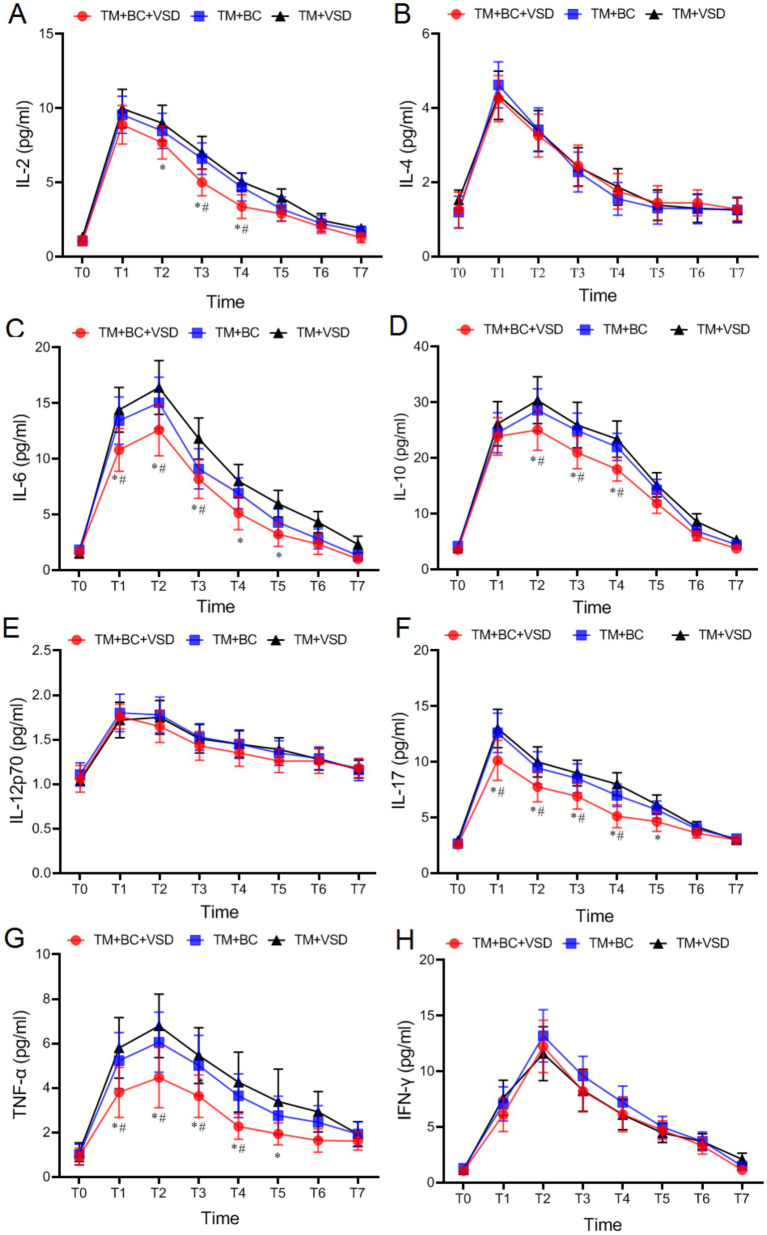
Serum inflammatory factor levels comparison among three goat groups. **(A)** IL-2 comparison among the three groups; **(B)** IL-4 comparison among the three groups; **(C)** IL-6 comparison among the three groups; **(D)** IL-10 comparison among the three groups; **(E)** IL-12p70 comparison among the three groups; **(F)** IL-17 comparison among the three groups; **(G)** TNF-*α* comparison in three groups; **(H)** IFN-*γ* comparison among the three groups of goat. Compared with the TM + VSD group, ^*^p < 0.05; Compared with the TM + BC group, ^#^*p* < 0.05.

There were no statistically significant differences in the levels of IL-6, IL-2, IL-10, IL-17, IL-4, IL-12p70, TNF-*α* and IFN-*γ* among the three goat groups before the operation (*p* > 0.05, Figure 7). All measured indices initially increased and then decreased after the operation (*p* < 0.05, Figure 7). The levels of IL-2 and IL-10 at T2–T4, and IL-6, IL-17 and TNF-α at T1–T5 in the TM + BC + VSD group were significantly lower than those in the TM + VSD group. The levels of IL-2 at T3–4, IL-6 at T1–3, IL-10 at T2–T4, IL-17 and TNF-α at T1–T4 were significantly lower than those in the TM + BC group (*p* < 0.05, Figure 7). No statistically significant differences were observed in the remaining indicators among the three groups (*p* > 0.05, Figure 7).

## 4 Discussion

The chest wall defects caused by bullets and explosions during wartime, as well as the extensive tissue defects remaining after chest wall surgery due to trauma, infection, or other reasons, disrupt the integrity of the thoracic cage. This leads to chest wall softening and abnormal breathing, resulting in respiratory and circulatory dysfunction. Repair and treatment are challenging, and the mortality rate is high (14, 15). With the increase in chest trauma and chest wall diseases, clinical cases requiring thoracic reconstruction have gradually risen, garnering growing attention from thoracic surgeons.

At present, materials for reconstructing chest wall defects are classified into three types: autologous tissues, allogeneic tissues and artificial materials. Reconstructing the bony thorax using a patient’s own tissues has disadvantages such as surgical complexity, significant trauma, and limited material availability, making it unsuitable for early or rapid thoracic reconstruction (16, 17). While tissue-engineered bone offering advantages like high histocompatibility, *in vivo* degradation and replacement, and no need for removal surgery, its limited supply, high cost, prolonged osteogenesis process, and potential immune rejection still restrict its clinical application (18–20). Artificial biosynthetic materials (e.g., Marlex patches, Prolene mesh, Gore-Tex patches, and Bard patches) exhibit high durability, good histocompatibility, no carcinogenicity and no interference from X-ray examination. However, postoperative complications such as respiratory distress, mesh detachment, sinus tract formation and infection remain common (21–23). Given the limitations of a single-material approaches, some scholars have used Hybrid techniques, first reconstructing the bony thorax and then applying patch materials to close chest wall defects. However, this method still has drawbacks, including restricted chest wall mobility, potential local effusion, infection, support structure loosening, and inapplicability in infected cases (24). Therefore, developing a more physiologically compatible method for bony thoracic reconstruction remains a key research focus in thoracic surgery.

In recent years, advancements in materials science and surgical techniques have significantly progressed chest wall reconstruction. Notably, the application of titanium alloys (TM) (8–10, 25), BC (26, 27) and VSD technology (28) has introduced new possibilities for repairing chest wall defects. Yang et al. (29) results showed that chest wall reconstruction utilizing synthetic TM following extensive resections of the malignant tumors of the chest wall allowed for adequate resection size, with acceptable complications and survival benefits. Jung et al. (30) results showed that BC blocks with rigid plate fixation systems are cost-effective alternatives for sternal reconstruction following sternal resection. Gabriel et al. (31) reported that VSD facilitated positive healing outcomes in patients with deep sternal wound infections after sternal defect reconstruction following cardiothoracic surgery. However, these materials or techniques exhibit clear limitations when used alone. TM stabilizes the thoracic cage but fails to address open pneumothorax or wound infections caused by its porous structure. BC reduces infection risk but lacks structural support and is unable to prevent chest wall flail or provide adequate drainage. VSD seals the thoracic cavity and ensures drainage, but cannot correct abnormal respiration from bony defects. Current research has confirmed that the antibiotic-loaded BC combined with VSD may be an effective method for the sternal reconstruction of deep sternal wound infections and can improve the patient’s lung function in a short time (32). In addition, research findings showed that the reconstruction of chest wall defects with mesh, BC, and a titanium rib plate system was an appropriate method to prevent instability of the chest wall (33). However, there are no relevant studies on the combined application of the three methods at present. Therefore, this study pioneers a composite layered repair system, where materials with distinct properties are surgically layered into a functional complex for chest wall reconstruction. Using a goat chest wall defect model, we analyzed this system’s efficacy by combining TM, BC and VSD. The results showed that the TM + BC + VSD group exhibited the most excellent repair effect, and its survival rate (85.7%) was higher than that of the TM + VSD group (71.4%). The analysis of the reasons might be that the continuous negative pressure aspiration of VSD effectively reduces wound effusion, while the local sustained-release effect of antibiotic BC significantly reduces the risk of infection. Notably, all deceased animals developed severe pulmonary infections or respiratory failure, suggesting that special attention should be paid to the prevention of postoperative pulmonary complications in clinical applications. Meanwhile, the TM + BC + VSD group showed significant advantages in functional recovery, and its activity status score was significantly better than that of the TM + VSD group in the early postoperative period and significantly better than that of the TM + BC group in the middle recovery period. Those indicated that the composite layered repair system may promote functional recovery through multiple mechanisms: TM provides structural support, BC maintains chest wall stability, and VSD optimizes the local microenvironment. Furthermore, the superior performance of the TM + BC + VSD group in oxygenation indicators such as SpO_2_ and hemoglobin may be related to its better maintenance of chest wall compliance, which reduces the occurrence of restrictive ventilation dysfunction.

Studies have shown that BC filled in the bone defect area can stimulate surrounding soft tissues to form an induction membrane and enhancing stability (34, 35). The closed space created by this induction membrane prevents external inflammatory factors and bacteria from infiltrating the defect site, thereby reducing infection risk (36, 37). VSD can mitigate wound infection and down-regulate pro-inflammatory factor expression in wound tissue (38). Bassetto et al. (39) demonstrated that VSD’s effects extend beyond superficial granulation tissue to deeper structures, alleviating inflammation and promoting tissue stabilization. Other studies suggest that VSD treatment modulates cytokine and growth factor profiles in wounds via mechanoreceptor and chemoreceptor signaling, shifting the balance toward anti-inflammatory responses (40). Recent studies have found that the combined treatment of antibiotic-loaded BC and VSD for MDROs-DFUs not only significantly shortens hospital stays and the time to achieve negative MDROs but also reduces patients’ pain and burden, while promoting postoperative recovery, improving local blood supply, effectively reducing inflammatory reactions, and accelerating wound healing (41). In this study, we analyzed the impact of the composite layered repair system (TM + BC + VSD) on inflammatory cytokines. The results showed that all animals experienced a typical post-traumatic inflammatory response process (peaking followed by decline), but the TM + BC + VSD group showed lower peak inflammation and faster recovery. Moreover, the expression levels of multiple pro-inflammatory factors (e.g., IL-6, IL-17, TNF-*α*) of the TM + BC + VSD group were significantly lower than those of the other two groups. We speculate that this might stem from the local anti-infective effect of antibiotic BC, VSD reduces the retention of necrotic tissue, and the mechanical stability of the composite structure alleviates secondary damage. These insights offer a novel perspective on the molecular mechanisms of trauma repair.

In conclusion, the composite layered repair system (TM + BC + VSD) demonstrates significant advantages for chest wall defect repair. It can better maintain the stability of vital signs in goat, promote wound recovery, effectively improve the blood routine and blood gas indicators of goat, reduce inflammatory responses, promote vascular formation, and thereby promote the regeneration of chest wall defect tissues. It has certain reference value for improving the success rate of treating thoracic war trauma and reducing the disability rate. This study has certain limitations, including that anatomical differences between animal models and humans may limit clinical extrapolation, the short observation period lacks long-term follow-up data, and the small sample size reduces statistical power, etc. Therefore, subsequent studies can extend the observation period to more than 6 months and conduct in-depth mechanism exploration in combination with molecular biological detection methods such as Western blot and PCR.

## Data Availability

The original contributions presented in the study are included in the article/supplementary material, further inquiries can be directed to the corresponding author/s.
